# The Association Between Intimate Partner Violence and Anemia Among Ethiopian Women in Their Reproductive Age (15–49 Years): Analysis of National Survey Data

**DOI:** 10.1155/bmri/6321439

**Published:** 2025-09-22

**Authors:** Tenaw Yimer Tiruye, Emebet Gashaw Wassie

**Affiliations:** ^1^ Allied Health and Human Performance Unit, University of South Australia, Adelaide, South Australia, Australia, unisa.edu.au; ^2^ Independent Researcher, Adelaide, South Australia, Australia

**Keywords:** intimate partner violence, partner control, nutrition, anemia, gender and nutrition

## Abstract

**Background:** In Ethiopia, there is a high prevalence of intimate partner violence (IPV) and anemia, yet there is limited empirical evidence on the potential link between these two public health problems. This study was aimed at investigating the potential association between IPV and anemia among Ethiopian women aged 15–49 years old.

**Methods:** We used secondary data from the 2016 Ethiopian Demographic and Health Survey (EDHS). All women who participated in the domestic violence module of the EDHS were studied. The exposure variable was the experience of physical, sexual, or emotional violence, as well as controlling behaviors, perpetrated by a current or former partner. Anemia was defined as a hemoglobin level of < 11.0 g/dL for pregnant women and < 12.0 g/dL for nonpregnant women. Multilevel binomial logistic regression models, which accounted for EDHS’s hierarchical data structure and adjusted for demographic, socioeconomic, and fertility‐related factors, were applied.

**Results:** Among the 4265 individuals included in the analysis, 1081 (25.4%) women had anemia. Notably, 64.1% of women have experienced at least one form of IPV, while 13.1% have experienced multiple forms of IPV. Significant associations were observed between IPV and anemia (adjusted odds ratio [AORs] 1.39, 95% confidence interval [CI]: 1.09–1.78). Furthermore, the odds of anemia were 1.6 times higher (AOR 1.61, 95% CI: 1.26–2.06) among women who had experienced multiple acts of IPV compared to those who had experienced none.

**Conclusion:** The association between IPV and anemia suggests the importance of integrating IPV screening tools and intervention strategies into women’s nutrition programs.

## 1. Background

Anemia, often referred to as a “hidden hunger” [1], is primarily caused by deficiencies in micronutrients, particularly iron and folate [2]. This condition has a significant global burden, affecting 27% of the global population, with 89% of cases occurring in developing countries [3]. In Ethiopia, approximately 24% of women suffer from anemia [4]. In some areas of Ethiopia, the prevalence is even higher, ranging from 36% to 44% [5–7]. Anemia has detrimental health implications for women and their children, contributing to preventable maternal mortality [4, 8], low birth weight [9], stillbirth [10], poor intellectual development [3], and increased susceptibility to infections [3].

Anemia arises from a complex interplay of biological and social factors. Biologically, deficiencies in essential nutrients like iron, vitamin B12, and folate are primary drivers, impacting red blood cell production and/or function [11]. Genetic predispositions and chronic diseases also play a role [12]. Socially, factors such as socioeconomic status, education levels, and cultural practices influence access to nutritious food and healthcare [13]. For women, nutritional health is further shaped by dietary habits, often influenced by societal norms and food availability [14]. Pregnancy significantly increases iron demands, making women particularly vulnerable to anemia [15]. Moreover, access to quality healthcare services, including prenatal care and nutritional counseling, is crucial for the prevention and management of anemia [11].

There is an emerging interest in understanding the potential impact of psychological stressors, such as intimate partner violence (IPV), on women’s nutritional status. Previous studies from India [16] and Nepal [17] have demonstrated a link between IPV and anemia. Research specifically investigating the direct correlation between IPV and anemia in African women is limited. A study conducted in Nigeria found a higher prevalence of anemia among women in rural areas who reported experiencing food‐based domestic violence [18]. This could be due to several factors, including the physical injuries and nutritional deficiencies resulting from violence, the psychological stress impacting appetite and nutrient absorption, and limited access to healthcare and adequate nutrition due to controlling behaviors by partners.

There is extensive evidence showing that IPV increases the likelihood of experiencing mental health issues, such as stress and substance abuse [19–22], and diminishes individuals’ autonomy in making decisions about their reproductive health [23–25]. These health problems and health behaviors related to IPV, such as substance abuse [7, 26], impaired decision‐making regarding fertility [6, 7, 27–29], failure to use contraception [6, 26], mental disorders [30, 31], and stress [32–34], are known to increase the risk of anemia. This implies that it is reasonable to hypothesize that women’s experience of IPV could be associated with anemia.

Studies conducted in Ethiopia have shown that several factors are associated with anemia. These factors include the lack of iron supplementation [5, 35], inadequate dietary intake [5], consumption of tea immediately after meals [36], limited dietary diversity [37], adherence to food taboos during pregnancy [38], infections such as malaria [6, 36], and substance abuse [7, 26]. Fertility‐related factors, such as nonuse of contraception [6, 26], number of births [6, 7, 27, 35, 37], and termination of pregnancy [27], are also significant factors that increase the likelihood of anemia. However, none of the existing studies have explored the potential link between IPV and women’s overall nutrition, specifically anemia. Given the high prevalence of both IPV (up to 71% [39]) and anemia in Ethiopia, it is crucial to investigate the association between these two public health issues. Therefore, the aim of this study was to investigate the association between different forms of IPV and anemia among women in Ethiopia. This study builds upon a previous work [40], which involved a broader investigation of the impact of IPV.

## 2. Methods

### 2.1. Data Source and Sampling

Data from the 2016 Ethiopian Demographic and Health Survey (EDHS) was utilized for this study. The 2016 EDHS was a nationwide cross‐sectional survey conducted from January 18 to June 27, 2016 [4]. A two‐stage stratified cluster sampling method was employed to select participants. Initially, 645 clusters were selected, comprising 202 clusters from urban areas and 443 clusters from rural areas, based on proportional‐to‐size allocation. Subsequently, 28 households were selected per cluster using systematic random sampling. In total, 15,683 women aged 15–49 years participated in the general survey [4]. For the substudy on domestic violence, only one married woman per household participated, resulting in 5860 women being interviewed. For the purpose of this analysis, we included ever‐married women who had complete data on their blood hemoglobin levels and responded to the IPV questionnaire. A total of 4265 women were included in our analyses.

### 2.2. Measurement and Variables

The dependent variable, anemia, was defined as hemoglobin levels of < 11.0 g/dL for pregnant women and < 12.0 g/dL for nonpregnant women. Blood samples were drawn from a drop of blood taken from a finger prick and collected in a microcuvette. Hemoglobin analysis was carried out on‐site using a battery‐operated portable HemoCue analyzer [4]. The hemoglobin levels were then adjusted for cigarette smoking and altitude (in areas > 1000 m).

The primary independent variable was IPV (physical, emotional, sexual, or partner control). In the EDHS, IPV was measured based on women’s self‐reporting of the number of violent acts they had experienced in their relationships throughout their lifetime. This includes acts perpetrated by husbands or partners against currently married women and acts perpetrated by most recent husbands or partners against previously married women. IPV was categorized as “yes” if women had experienced any of the 18 acts from a partner in their lifetime and “no” if they had not. The tool used to assess IPV in EDHS 2016 was “did your partner/husband…”

1. Push you, shake you, or throw something at you?
2. Slap you?
3. Twist your arm or pull your hair?
4. Punch you with his/her fist or with something that could hurt you?
5. Kick you, drag you, or beat you up?
6. Try to choke you or burn you on purpose?
7. Threaten or attack you with a knife, gun, or any other weapon?
8. Physically force you to have sexual intercourse with him even when you did not want to?
9. Physically force you to perform any other sexual acts you did not want to?
10. Force you with threats or in any other way to perform sexual acts you did not want to?
11. Say or do something to humiliate you in front of others?
12. Threaten to hurt or harm you or someone close to you?
13. Insult you or make you feel bad about yourself?
14. Being jealous if you talk to men
15. Accusing you of being unfaithful
16. Does not allow you to meet your friends
17. Limits your contact with family
18. Tries to know where you are at all times

Questions 1–7 measure physical spousal violence, Questions 8–10 measure sexual IPV, Questions 11–13 measure emotional IPV, and Questions 14–18 measure partner controlling acts.

Additionally, we examined the relationship between IPV and anemia by categorizing IPV based on the number of violent acts (i.e., single act vs. multiple acts), with an aim to test whether there is a “dose–response” gradient effect of the number of violent acts on the occurrence of anemia.

Potential confounding variables that needed to be controlled in order to examine the association between the exposure (IPV) and the outcome (anemia) were identified from previous literature [6, 7, 16, 26, 27, 40–46]. These variables were deemed to be associated with both the exposure (IPV) [40, 43–46] and outcome (anemia) [6, 7, 16, 26, 27, 40–42] and hence play a potential confounding role. The definitions and categories of these variables are presented in File S1.

### 2.3. Statistical Analyses

A series of multilevel binomial logistic regression models were executed to consider the composite IPV measure and each of the IPV forms separately, with each model including one IPV exposure domain at a time. The multilevel models accounted for the hierarchical nature of the EDHS data, where women are nested within clusters. The models were adjusted for various factors including age, age at first marriage, educational attainment, employment status, residence (urban/rural), region of residence, decision‐making autonomy, contraceptive use, body mass index (BMI), number of children ever born, substance abuse, socioeconomic status, and access to media. All analyses considered the EDHS sampling weights and were based on the weighted sample. The sampling weights used in the EDHS accounted for the complex sampling procedures (multistage stratified cluster sampling) that might cause an unequal probability of selection for certain areas or subgroups either due to design or coincidence. Hence, sampling weights were adjusted for differences in probability of selection and interview to allow extrapolation of results to the national level of representativeness [47]. The Akaike information criterion (AIC) and likelihood ratio tests were employed to compare and select the models that best fit the data. Finally, the results from the final models with the best fit were reported. Adjusted odds ratios (AORs), along with their corresponding 95% CI, were used to report associations. A *p* value less than 0.05 is considered statistically significant.

### 2.4. Ethics Approval

This study utilized publicly available EDHS 2016 data, which did not contain personally identifiable information. As the data is in the public domain, ethical approval was not required. However, permission to use the data was obtained from the DHS program (https://dhsprogram.com/Data) following online registration prior to analysis.

## 3. Results

The majority of the study participants fell within the 25–29 age group (22.1%), were married before the age of 18 (63.3%), had no formal education (61.3%), and resided in rural areas (82.6%). A significant proportion of participants (40.1%) lacked decision‐making autonomy, and 42.6% had never used any form of contraception. Additionally, 49.1% of individuals reported a history of substance abuse, and 62.9% had no access to media (Table 1).

**Table 1:** Participant characteristics, EDHS, 2016 (*n* = 4265).

**Variable**	**Class**	**Weighted frequency** ^**a**^	**Weighted percentage** ^**b**^
Current age	15–19	271	6.4
	20–24	650	15.3
	25–29	943	22.1
	30–34	875	20.5
	35–39	688	16.1
	40–44	476	11.2
	45–49	363	8.5

Age at first cohabitation	< 15 years	1116	26.2
	15–18 years	1582	37.1
	≥ 18 years	1566	36.7

Educational status	No education	2613	61.3
	Primary	1186	27.8
	Secondary and Above	466	10.9

Employment status	Not employed	2041	47.9
	Employed	2224	52.2
Type of place of residence	Urban	743	17.4
	Rural	3522	82.6
Region of residence	Tigray	302	7.1
	Afar	41	1.0
	Amhara	1076	25.2
	Oromia	1628	38.2
	Somali	124	2.9
	Benishangul	40	1.0
	SNNPR	882	20.7
	Gambela	12	0.3
	Harari	8	0.2
	Addis Ababa	132	3.1
	Dire Dawa	20	0.5

Decision‐making autonomy	No	1709	40.1
	Yes	2556	59.9
Contraceptive use	Never used	1816	42.6
	Used	2449	57.4
BMI (kg/m^2^)	Not undernutrition	3408	79.9
	Undernutrition	857	20.1
Number of children ever born	One or less	641	15.0
	2–3	1147	26.9
	4–5	942	22.1
	> 5	1535	36.0

Substance abuse	No	2171	50.9
	Yes	2094	49.1
Wealth index	Poorest	801	18.8
	Poorer	829	19.4
	Middle	905	21.2
	Richer	824	19.3
	Richest	906	21.2

Access to media	No access	2683	62.9
	< once a week	635	14.9
	≥ once a week	947	22.2

*Note:* The sampling weights were used to adjust for the unequal probability of selection in EDHS and to make the results nationally representative.

Abbreviations: BMI, body mass index; SNNPR, Southern Nations, Nationalities, and Peoples’ Region.

^a^Numbers may not total 4265 due to rounding.

^b^Percentages may not total 100% due to rounding.

Table 2 presents the estimated prevalence of various forms of IPV with a 95% CI. Sexual IPV was the least prevalent form, accounting for 11.9% of cases, while partner control was the most prevalent, accounting for 56.7% of cases. One in every four women has experienced emotional IPV, and two in every three women have encountered at least one form of IPV. About 13% of women have experienced more than two types of IPV. The prevalence of anemia among women was 25.4% (95% CI: 22.9–27.8).

**Table 2:** Prevalence of the different forms of IPV against women, EDHS, 2016 (*n* = 4265).

**Form of IPV**	**Prevalence (%)**	**95% CI**
Physical IPV	25.1	(22.8, 27.3)
Sexual IPV	11.9	(10.1, 13.7)
Emotional IPV	24.1	(21.7, 26.5)
Partner controlling behavior (single form)	56.7	(53.8, 59.7)
Any form of IPV	64.1	(57.4, 70.9)
Multiple types of violence (two or more)	13.1	(10.6, 15.6)

Abbreviations: CI, confidence interval; IPV, intimate partner violence.

Upon adjusting for potential confounders, a significant association was found between experiencing any form of IPV (physical, sexual, emotional, and/or partner control) and anemia (AOR 1.39, 95% CI: 1.09–1.78). Similarly, experiencing multiple acts of IPV was significantly associated with anemia (AOR 1.61, 95% CI: 1.26–2.06). Emotional IPV also exhibited a significant positive association with anemia (AOR 1.40, 95% CI: 1.02–1.93) (Table 3).

**Table 3:** Associations between IPV and anemia among women, EDHS, 2016.

**IPV forms**	**Category**	**Participant was anemic**	
		**AOR (95% CI)** ^**a**^	**p** **value**
Physical IPV	No	1.00	
	Yes	1.41 (0.87–2.35)	0.102
Sexual IPV	No	1.00	
	Yes	1.18 (0.91, 1.53)	0.210
Emotional IPV	No	1.00	
	Yes	1.40 (1.02, 1.93)	0.037
Partner controlling behavior	No	1.00	
	Yes	1.34 (0.99, 1.83)	0.062
IPV (all)^b^	No	1.00	
	Yes	1.39 (1.09, 1.78)	0.009
IPV (all)^c^	No	1.00	
	Single	1.23 (0.88, 1.57)	0.144
	Multiple	1.61 (1.26, 2.06)	< 0.001

*Note:* 1.00, reference category.

Abbreviations: AOR, adjusted odds ratio; CI, confidence interval; IPV, intimate partner violence.

^a^Adjusted for age, age at first marriage, educational attainment, employment status, rurality (urban/rural), region of residence, decision‐making autonomy, contraceptive use, BMI, number of children ever born, substance abuse, wealth, and access to media.

^b^“Yes” if women reported experiencing at least one of the four IPV forms.

^c^We further assessed the association to test the hypothesis that the concentration of violent acts (experiencing multiple forms of violence) has a more significant effect on anemia than any single act.

## 4. Discussion

This study, which is built upon a previous work [40], identified a significant positive association between women’s experience of any form of IPV and anemia. This association remained statistically significant even after adjusting for potential confounding variables. A notable aspect of this study is its examination of each form of IPV individually, as well as IPV as a composite measure.

Our results are consistent with earlier studies conducted in other developing countries such as Nigeria [18], India [16], Bangladesh [41], and Nepal [17]. A recent investigation of the relationship between food‐based violence and anemia among Nigerian women showed that anemia was 29% higher among rural women who approved food‐based domestic violence (AOR 1.29, 95% CI: 1.15–1.57) than among those who did not. In this same study, food‐based domestic violence was not significantly associated with anemia among urban women [18]. A study from India showed a positive association of multiple incidents of domestic violence in the previous year with anemia (AOR 1.11, 95% CI: 1.04–1.18) and underweight (AOR 1.21, 95% CI: 1.13–1.29) [16], while a study from Bangladesh found that experiencing physical IPV, either alone or in conjunction with sexual IPV, increased the likelihood of undernutrition among women (physical IPV alone, AOR 1.22, 95% CI: 1.02–1.46 and both physical and sexual IPV, AOR 1.24, 95% CI: 1.04–1.58) [41]. Another study from Nepal has shown that lifetime experience of controlling behavior (AOR 1.25, 95% CI: 1.03–1.53) was associated with an increased risk of anemia [17]. According to this study, several other forms of IPV such as low intensity of emotional IPV (both recent and lifetime experience) and moderate intensity of physical IPV experienced in the preceding year were associated with an increased risk of overweight/obesity, while moderate intensity of sexual IPV (AOR 2.59, 95% CI: 1.10–6.11) experienced in the preceding year was associated with an increased risk of underweight BMI [17].

Different potential pathways can be proposed to explain the observed associations between IPV and anemia. These pathways are likely interconnected and may operate simultaneously to influence women’s overall health and nutritional status. One significant pathway is the nutritional impact of IPV, where women experiencing IPV may face reduced access to food, controlling behaviors exerted by their partners regarding food consumption, and a consequent lack of dietary diversity. Food‐based violence, as observed in the Nigerian study [18], directly restricts a woman’s ability to meet her nutritional needs, increasing the risk of iron deficiency and other micronutrient deficiencies that can lead to anemia. Furthermore, IPV can contribute to eating disorders, unhealthy weight loss, and overall food insecurity [48], all of which can negatively impact a woman’s nutritional status and increase her susceptibility to anemia. Physical injuries resulting from IPV represent another potential pathway. While perhaps less direct than nutritional impact, the potential for blood loss due to physical violence could contribute to the development or exacerbation of anemia. Repeated or severe injuries could lead to chronic blood loss, depleting iron stores and lowering hemoglobin levels. The psychological stress pathway associated with experiencing violence can significantly influence a woman’s eating behaviors and appetite, metabolism and nutrient absorption, and overall mental health such as depression and anxiety, which can further increase the likelihood of developing anemia [16, 30, 31, 49].

Women in abusive relationships often face limitations in decision‐making regarding matters such as healthcare access [50–52], fertility control [28, 29, 40, 53–56], and food selection and preparation [16, 30, 49] due to coercive control by their partners. In Ethiopia, pregnant women attending prenatal care are often provided with nutritional counseling to modify their dietary behaviors [26, 57]. However, restricted access to healthcare by abusive partners may lead to a lack of receiving nutritional counseling [30]. Additionally, factors such as the number of births [6, 7, 27], lack of contraceptive use [6, 26], and termination of pregnancy [27] are identified as significant contributors to anemia among Ethiopian women. Reproductive coercion and fertility‐related issues are commonly encountered by women who have experienced abuse [23–25, 28, 29, 40, 56].

Another theory is that women’s mental health and health behaviors are influenced by IPV. It has been widely investigated that IPV is associated with various mental health problems, including depression, anxiety, stress, posttraumatic stress disorder (PTSD), and eating and sleeping disorders [19, 20]. Mental health problems can further lead to negative behaviors, such as alcohol and drug abuse [19–22], as well as hindering their ability to adopt healthy lifestyles [20]. Women who engage in negative health behaviors, such as substance abuse, are prone to exhibit restrictive dietary behaviors [7]. In Ethiopia, women who chewed khat (a green leaf consumed as a stimulant) were more likely to experience anemia compared to nonkhat chewers [7, 26]. The relationship between mental health disorders and nutritional anemia has also been explored [30, 31]. Stress triggered by IPV [32–34] potentially leads to eating and metabolic disorders [16, 49]. This is reflected in our study, where emotional IPV demonstrated a significant positive association with anemia (AOR 1.40, 95% CI: 1.02–1.93). Hence, anemia associated with IPV may not only be nutritional but also hemolytic anemia caused by oxidative stress, which causes premature destruction of red blood cells before their normal lifespan. Overall, there is a need for future research to investigate causal pathways in the observed relationships between IPV and anemia. This research provided some theoretical foundation for further research.

This study presents the first national evidence establishing a potential link between different forms of IPV and anemia among women. The evidence is derived from nationally representative and high‐quality data collected by well‐trained data collectors using standardized measurement tools. The identified association between IPV and anemia offers valuable insights for healthcare program planners concerned with maternal health, nutrition, and IPV. The findings suggest that in Ethiopia, where anemia is prevalent, IPV imposes an additional burden that could lead to increased healthcare costs for society. Therefore, comprehensive strategies for preventing IPV, including interventions and screening tools integrated into women’s nutrition programs, are necessary.

The study has the following limitations. First, while we accounted for various socioeconomic, demographic, and fertility‐related factors in our models, we did not adjust for clinical factors, such as coexisting chronic diseases, mental health conditions, and infections, that were not available in our datasets. Due to the complex pathophysiological processes involved in the development of anemia, our findings may be influenced by unmeasured variables. Second, our cross‐sectional study design makes it difficult to establish causal relationships. However, given that the outcome of interest (anemia) was measured during the survey and the exposure (IPV) was captured retrospectively (i.e., before the survey), it is plausible to establish a temporal order for the association between IPV and anemia. Third, underreporting of IPV may occur due to social desirability and recall biases. Nevertheless, EDHS follows standard procedures and precautions to minimize the chances of underreporting. This includes asking multiple questions to accurately assess IPV and providing thorough training to data collectors. Finally, while acknowledging the time elapsed since the 2016 EDHS, its comprehensive and nationally representative nature still offers a unique advantage over smaller, localized studies, providing a robust baseline understanding of the possible link between IPV and anemia in Ethiopia. While advancements and changes in the landscape are likely to have occurred since 2016, this study establishes a necessary foundation upon which future research using more recent data, when available, can build.

## 5. Conclusion

The observed association between IPV and anemia among Ethiopian women underscores the importance of integrating IPV interventions into women’s health and nutrition programs. This integration has the potential to mitigate adverse health consequences linked to anemia in women and children. Given that women in abusive relationships may be reluctant to seek healthcare, it is recommended that nutrition program planners create home‐based support strategies for victims.

**Nomenclature**
AOR: adjusted odds ratio
BMI: body mass index
CI: confidence interval
EDHS: Ethiopian Demographic and Health Survey
IPV: intimate partner violence

## Disclosure

Both authors agreed on the submission of the manuscript.

## Conflicts of Interest

The authors declare no conflicts of interest.

## Author Contributions

Both authors are involved from the conception of the study to the preparation of the manuscript.

## Funding

No funding was received for this manuscript.

## Supporting information


**Supporting Information File S1** Additional supporting information can be found online in the Supporting Information section. summarizes the definitions and categories of potential confounding variables adjusted in our multivariable models to assess the independent association between intimate partner violence and anemia.

## Data Availability

The data that support the findings of this study are publicly available from the Demographic and Health Survey program. The data can be accessed upon online registration and approval through https://dhsprogram.com/data/new-user-registration.cfm.

## References

[1] Wondu T. and Bijlsma M. , “The Hidden Hunger”: Understanding the Burden of Anaemia and Its Determinants Among Pregnant and Non-Pregnant Women in Ethiopia, African Journal of Food, Agriculture, Nutrition and Development. (2012) 12, no. 7, 10.18697/ajfand.55.11015.

[2] Kaur M. , Chauhan A. , Manzar M. D. , and Rajput M. M. , Maternal Anaemia and Neonatal Outcome: A Prospective Study on Urban Pregnant Women, Journal of Clinical and Diagnostic Research. (2015) 9, no. 12, QC04-8, 10.7860/JCDR/2015/14924.6985, 2-s2.0-84949469807. PMC4717682 26816949

[3] Kassebaum N. J. , Jasrasaria R. , Naghavi M. , Wulf S. K. , Johns N. , Lozano R. , Regan M. , Weatherall D. , Chou D. P. , Eisele T. P. , Flaxman S. R. , Pullan R. L. , Brooker S. J. , and Murray C. J. L. , A Systematic Analysis of Global Anemia Burden From 1990 to 2010, Blood. (2014) 123, no. 5, 615–624, 10.1182/blood-2013-06-508325, 2-s2.0-84894067309, 24297872. 24297872 PMC3907750

[4] Central Statistical Agency (CSA) [Ethiopia], ICF. Ethiopia Demographic and Health Survey 2016. *Addis Ababa, Ethiopia, and Rockville, Maryland*, USA: CSA and ICF; 2017, https://dhsprogram.com/pubs/pdf/fr328/fr328.pdf.

[5] Gebre A. and Mulugeta A. , Prevalence of Anemia and Associated Factors Among Pregnant Women in North Western Zone of Tigray, Northern Ethiopia: A Cross-Sectional Study, Journal of Nutrition and Metabolism. (2015) 2015, no. 1, 165430, 10.1155/2015/165430, 2-s2.0-84934903055, 26137321. 26137321 PMC4475559

[6] Gedefaw L. , Ayele A. , Asres Y. , and Mossie A. , Anemia and Associated Factors Among Pregnant Women Attending Antenatal Care Clinic in Wolayita Sodo Town, Southern Ethiopia, Ethiopian Journal of Health Sciences. (2015) 25, no. 2, 155–164. 26124623 10.4314/ejhs.v25i2.8 PMC4478267

[7] Kedir H. , Berhane Y. , and Worku A. , Khat Chewing and Restrictive Dietary Behaviors Are Associated With Anemia Among Pregnant Women in High Prevalence Rural Communities in Eastern Ethiopia, PLoS ONE. (2013) 8, no. 11, e78601, 10.1371/journal.pone.0078601, 2-s2.0-84892589963. 24223828 PMC3817221

[8] Tinker A. , Women′s Health: The Unfinished Agenda, International Journal of Gynaecology and Obstetrics. (2000) 70, no. 1, 149–158, 10.1016/S0020-7292(00)00227-7, 2-s2.0-0034237635. 10884543

[9] Elhassan E. M. , Abbaker A. O. , Haggaz A. D. , Abubaker M. S. , and Adam I. , Anaemia and Low Birth Weight in Medani, Hospital Sudan, BMC Research Notes. (2010) 3, no. 1, 10.1186/1756-0500-3-181, 2-s2.0-77954978167, 20584294. PMC2907404 20584294

[10] Maghsoudlou S. , Cnattingius S. , Stephansson O. , Aarabi M. , Semnani S. , Montgomery S. M. , and Bahmanyar S. , Maternal Haemoglobin Concentrations Before and During Pregnancy and Stillbirth Risk: A Population-Based Case-Control Study, BMC Pregnancy and Childbirth. (2016) 16, no. 1, 10.1186/s12884-016-0924-x, 2-s2.0-84973176757, 27259282. PMC4893297 27259282

[11] World Health Organization (WHO) , Anemia in Women and Children , 2025, [Available from: https://www.paho.org/en/enlace/anemia-women-and-children.

[12] Brittenham G. M. , Moir-Meyer G. , Abuga K. M. , Datta-Mitra A. , Cerami C. , Green R. , Pasricha S. R. , and Atkinson S. H. , Biology of Anemia: A Public Health Perspective, Journal of Nutrition. (2023) 153, S7–S28, 10.1016/j.tjnut.2023.07.018, 37778889. 37778889 PMC7618251

[13] Habib N. , Abbasi S.-U.-R. S. , and Aziz W. , An Analysis of Societal Determinant of Anemia Among Adolescent Girls in Azad Jammu and Kashmir, Pakistan, Anemia. (2020) 2020, no. 1, 1628357, 10.1155/2020/1628357. 32047664 PMC7007924

[14] Ali S. A. , Abbasi Z. , Shahid B. , Moin G. , Hambidge K. M. , Krebs N. F. , Westcott J. E. , McClure E. M. , Goldenberg R. L. , and Saleem S. , Prevalence and Determinants of Anemia Among Women of Reproductive Age in Thatta Pakistan: Findings From a Cross-Sectional Study, PLoS One. (2020) 15, no. 9, e0239320, 10.1371/journal.pone.0239320, 32970719. 32970719 PMC7514090

[15] Achebe M. M. and Gafter-Gvili A. , How I Treat Anemia in Pregnancy: Iron, Cobalamin, and Folate, Blood. (2017) 129, no. 8, 940–949, 10.1182/blood-2016-08-672246, 2-s2.0-85015248244, 28034892. 28034892

[16] Ackerson L. K. and Subramanian S. V. , Domestic Violence and Chronic Malnutrition Among Women and Children in India, American Journal of Epidemiology. (2008) 167, no. 10, 1188–1196, 10.1093/aje/kwn049, 2-s2.0-43249106837, 18367471. 18367471 PMC2789268

[17] Chaudhary A. , Nakarmi J. , and Goodman A. , Association Between Intimate Partner Violence and Nutritional Status of Married Nepalese Women, Global Health Research and Policy. (2022) 7, no. 1, 10.1186/s41256-022-00248-0, 35585625. PMC9118640 35585625

[18] Ajoseh S. M. , Sifat R. I. , and Whesu J. T. , Food-Based Domestic Violence and Anemia Among Women in Sexual Unions in Nigeria: The Effect of Urbanization, Journal of Public Health Policy. (2024) 45, no. 3, 523–536, 10.1057/s41271-024-00504-2, 38992219. 38992219 PMC11315663

[19] Black M. C. , Intimate Partner Violence and Adverse Health Consequences, American Journal of Lifestyle Medicine. (2011) 5, no. 5, 428–439, 10.1177/1559827611410265, 2-s2.0-84993813808.

[20] Dutton M. A. , Green B. L. , Kaltman S. I. , Roesch D. M. , Zeffiro T. A. , and Krause E. D. , Intimate Partner Violence, PTSD, and Adverse Health Outcomes, Journal of Interpersonal Violence. (2006) 21, no. 7, 955–968, 10.1177/0886260506289178, 2-s2.0-33744799763. 16731994

[21] Lee B. X. , Donnelly P. D. , Cohen L. , and Garg S. , Violence, Health, and the 2030 Agenda: Merging Evidence and Implementation, Journal of Public Health Policy. (2016) 37, no. Supplement 1, 1–12, 10.1057/s41271-016-0011-6, 2-s2.0-85014714533, 27638239. 27638239

[22] Emenike E. , Lawoko S. , and Dalal K. , Intimate Partner Violence and Reproductive Health of Women in Kenya, International Council of Nurses. (2008) 55, no. 1, 97–102, 10.1111/j.1466-7657.2007.00580.x, 2-s2.0-39149098759. 18275542

[23] Devries K. M. , Kishor S. , Johnson H. , Stöckl H. , Bacchus L. J. , Garcia-Moreno C. , and Watts C. , Intimate Partner Violence During Pregnancy: Analysis of Prevalence Data From 19 Countries, Reproductive Health Matters. (2010) 18, no. 36, 158–170, 10.1016/S0968-8080(10)36533-5, 2-s2.0-78649394690, 21111360. 21111360

[24] Grace K. T. and Anderson J. C. , Reproductive Coercion: A Systematic Review, Trauma, Violence & Abuse. (2018) 19, no. 4, 371–390, 10.1177/1524838016663935, 2-s2.0-85053600215, 27535921. PMC5577387 27535921

[25] Miller E. , Decker M. R. , McCauley H. L. , Tancredi D. J. , Levenson R. R. , Waldman J. , Schoenwald P. , and Silverman J. G. , Pregnancy Coercion, Intimate Partner Violence, and Unintended Pregnancy, Contraception. (2010) 81, no. 4, 316–322, 10.1016/j.contraception.2009.12.004, 2-s2.0-77949263117, 20227548. 20227548 PMC2896047

[26] Lakew Y. , Biadgilign S. , and Haile D. , Anaemia Prevalence and Associated Factors Among Lactating Mothers in Ethiopia: Evidence From the 2005 and 2011 Demographic and Health Surveys, BMJ Open. (2015) 5, no. 4, e006001, 10.1136/bmjopen-2014-006001, 2-s2.0-84928252923, 25872935. PMC4401847 25872935

[27] Alemu T. and Umeta M. , Reproductive and Obstetric Factors Are Key Predictors of Maternal Anemia During Pregnancy in Ethiopia: Evidence From Demographic and Health Survey (2011), Anemia. (2015) 2015, no. 1, 649815, 10.1155/2015/649815, 2-s2.0-84941710816, 26417454. 26417454 PMC4568321

[28] Tiruye T. Y. , Harris M. L. , Chojenta C. , Holliday E. , and Loxton D. , The Mediation Effect of Contraceptive Use and Women’s Autonomy on the Relationship Between Intimate Partner Violence and Unintended Pregnancy in Ethiopia, BMC Public Health. (2020) 20, no. 1, 10.1186/s12889-020-09514-7, 32938435. PMC7493352 32938435

[29] Tiruye T. Y. , Harris M. L. , Chojenta C. , Holliday E. , and Loxton D. , Intimate Partner Violence Against Women in Ethiopia and Its Association With Unintended Pregnancy: A National Cross-Sectional Survey, International Journal of Public Health. (2020) 65, no. 9, 1657–1667, 10.1007/s00038-020-01510-3, 33048193. 33048193

[30] Tran T. D. , Biggs B. A. , Tran T. , Casey G. J. , Hanieh S. , Simpson J. A. , Dwyer T. , and Fisher J. , Psychological and Social Factors Associated With Late Pregnancy Iron Deficiency Anaemia in Rural Viet Nam: A Population-Based Prospective Study, PLoS One. (2013) 8, no. 10, e78162, 10.1371/journal.pone.0078162, 2-s2.0-84886041716, 24167605. 24167605 PMC3805582

[31] Bodnar L. M. and Wisner K. L. , Nutrition and Depression: Implications for Improving Mental Health Among Childbearing-Aged Women, Biological Psychiatry. (2005) 58, no. 9, 679–685, 10.1016/j.biopsych.2005.05.009, 2-s2.0-27544501675, 16040007. 16040007 PMC4288963

[32] Ziaei S. , Frith A. L. , Ekstrom E. C. , and Naved R. T. , Experiencing Lifetime Domestic Violence: Associations With Mental Health and Stress Among Pregnant Women in Rural Bangladesh: The MINIMat Randomized Trial, PLoS One. (2016) 11, no. 12, e0168103, 10.1371/journal.pone.0168103, 2-s2.0-85006959997, 27992478. 27992478 PMC5167379

[33] Stampfel C. C. , Chapman D. A. , and Alvarez A. E. , Intimate Partner Violence and Posttraumatic Stress Disorder Among High-Risk Women: Does Pregnancy Matter?, Violence Against Women. (2010) 16, no. 4, 426–443, 10.1177/1077801210364047, 2-s2.0-77949316457, 20224113. 20224113

[34] Flanagan J. C. , Gordon K. C. , Moore T. M. , and Stuart G. L. , Women′s Stress, Depression, and Relationship Adjustment Profiles as They Relate to Intimate Partner Violence and Mental Health During Pregnancy and Postpartum, Psychology of Violence. (2015) 5, no. 1, 66–73, 10.1037/a0036895, 2-s2.0-84925753686. 25642352 PMC4310243

[35] Gebremedhin S. , Enquselassie F. , and Umeta M. , Prevalence and Correlates of Maternal Anemia in Rural Sidama, Southern Ethiopia, African Journal of Reproductive Health. (2014) 18, no. 1, 44–53, 24796168. 24796168

[36] Alemayehu A. , Gedefaw L. , Yemane T. , and Asres Y. , Prevalence, Severity, and Determinant Factors of Anemia Among Pregnant Women in South Sudanese Refugees, Pugnido, Western Ethiopia, Anemia. (2016) 2016, no. 1, 9817358, 10.1155/2016/9817358, 2-s2.0-85008881535. 28058116 PMC5183745

[37] Roba K. T. , O′Connor T. P. , Belachew T. , and O′Brien N. M. , Seasonal Variation in Nutritional Status and Anemia Among Lactating Mothers in Two Agro-Ecological Zones of Rural Ethiopia: A Longitudinal Study, Nutrition. (2015) 31, no. 10, 1213–1218, 10.1016/j.nut.2015.03.007, 2-s2.0-84942295206, 26238533. 26238533

[38] Vasilevski V. and Carolan-Olah M. , Food Taboos and Nutrition-Related Pregnancy Concerns Among Ethiopian Women, Journal of Clinical Nursing. (2016) 25, no. 19-20, 3069–3075, 10.1111/jocn.13319, 2-s2.0-85027954137, 27411855. 27411855

[39] Garcia-Moreno C. , Jansen H. A. F. M. , Ellsberg M. , Heise L. , and Watts C. H. , Prevalence of Intimate Partner Violence: Findings From the WHO Multi-Country Study on Women′s Health and Domestic Violence, Lancet. (2006) 368, no. 9543, 1260–1269, 10.1016/S0140-6736(06)69523-8, 2-s2.0-33749249923. 17027732

[40] Tiruye T. Y. , Intimate Partner Violence Against Women in Ethiopia: Determinants, Impacts, and Health Sector Response, 2020, The University of Newcastle, [PhD thesis by publication].

[41] Rahman M. , Nakamura K. , Seino K. , and Kizuki M. , Intimate Partner Violence and Chronic Undernutrition Among Married Bangladeshi Women of Reproductive Age: Are the Poor Uniquely Disadvantaged?, European Journal of Clinical Nutrition. (2013) 67, no. 3, 301–307, 10.1038/ejcn.2012.202, 2-s2.0-84875227921, 23232590. 23232590

[42] Pryer J. A. , Rogers S. , and Rahman A. , Factors Affecting Nutritional Status in Female Adults in Dhaka slums, Bangladesh, Social Biology. (2003) 50, no. 3-4, 259–269, 10.1080/19485565.2003.9989075, 16382815. 16382815

[43] Heise L. L. and Kotsadam A. , Cross-National and Multilevel Correlates of Partner Violence: An Analysis of Data From Population-Based Surveys, Lancet Global Health. (2015) 3, no. 6, e332–e340, 10.1016/S2214-109X(15)00013-3, 2-s2.0-84930324192, 26001577. 26001577

[44] Jansen H. A. F. M. , Nguyen T. V. N. , and Hoang T. A. , Exploring Risk Factors Associated With Intimate Partner Violence in Vietnam: Results From a Cross-Sectional National Survey, International Journal of Public Health. (2016) 61, no. 8, 923–934, 10.1007/s00038-016-0879-8, 2-s2.0-84983456846, 27554794. 27554794

[45] Tiruye T. Y. , Harris M. L. , Chojenta C. , Holliday E. , and Loxton D. , Determinants of Intimate Partner Violence Against Women in Ethiopia: A Multi-Level Analysis, PLoS One. (2020) 15, no. 4, e0232217, 10.1371/journal.pone.0232217, 32330193. 32330193 PMC7182270

[46] Yimer T. , Gobena T. , Egata G. , and Mellie H. , Magnitude of Domestic Violence and Associated Factors Among Pregnant Women in Hulet Ejju Enessie District, Northwest Ethiopia, Advances in Public Health. (2014) 2014, no. 1, 484897, 10.1155/2014/484897.

[47] Croft T. N. , Marshall A. M. J. , Allen C. K. , Arnold F. , Assaf S. , and Balian S. , Guide to DHS Statistics, 2018, 645, 292–303, https://dhsprogram.com/pubs/pdf/DHSG1/Guide_to_DHS_Statistics_DHS-7.pdf.

[48] Khanna A. , Singh J. P. , and Sharma N. , Association Between Intimate Partner Violence and Nutritional Status of Children: A Systematic Review and Mata-Analysis, medRxiv. (2021) 2021-02, 10.1101/2021.02.19.21252077.

[49] Chomat A. M. , Solomons N. W. , Koski K. G. , Wren H. M. , Vossenaar M. , and Scott M. E. , Quantitative Methodologies Reveal a Diversity of Nutrition, Infection/Illness, and Psychosocial Stressors During Pregnancy and Lactation in Rural Mam-Mayan Mother-Infant Dyads From the Western Highlands of Guatemala, Food and Nutrition Bulletin. (2015) 36, no. 4, 415–440, 10.1177/0379572115610944, 2-s2.0-84986571160, 26481796. 26481796

[50] Stöckl H. , Filippi V. , Watts C. , and Mbwambo J. K. , Induced Abortion, Pregnancy Loss and Intimate Partner Violence in Tanzania: A Population Based Study, BMC Pregnancy and Childbirth. (2012) 12, no. 1, 10.1186/1471-2393-12-12, 2-s2.0-84858863114, 22390254. PMC3311557 22390254

[51] Arcos E. , Uarac M. , Molina I. , Repossi A. , and Ulloa M. , Impact of Domestic Violence on Reproductive and Neonatal Health, Revista Médica de Chile. (2001) 129, no. 12, 1413–1424, 10.4067/S0034-98872001001200007, 12083060. 12083060

[52] Ibrahim Z. M. , Sayed Ahmed W. A. , El-Hamid S. A. , and Hagras A. M. , Intimate Partner Violence Among Egyptian Pregnant Women: Incidence, Risk Factors, and Adverse Maternal and Fetal Outcomes, Clinical and Experimental Obstetrics & Gynecology. (2015) 42, no. 2, 212–219, 10.12891/ceog1829.2015, 26054122. 26054122

[53] Sarkar N. N. , The Impact of Intimate Partner Violence on Women′s Reproductive Health and Pregnancy Outcome, Journal of Obstetrics and Gynaecology. (2008) 28, no. 3, 266–271, 10.1080/01443610802042415, 2-s2.0-45749102114, 18569465. 18569465

[54] Nguyen P. H. , Nguyen S. V. , Nguyen M. Q. , Nguyen N. T. , Keithly S. C. , Mai L. T. , Luong L. T. , and Pham H. Q. , The Association and a Potential Pathway Between Gender-Based Violence and Induced Abortion in Thai Nguyen Province, Vietnam, Global Health Action. (2012) 5, no. 1, 19006, 10.3402/gha.v5i0.19006, 23195517. PMC3511781 23195517

[55] Pallitto C. C. , Garcia-Moreno C. , Jansen H. A. , Heise L. , Ellsberg M. , and Watts C. , Intimate Partner Violence, Abortion, and Unintended Pregnancy: Results From the WHO Multi-Country Study on Women′s Health and Domestic Violence, International Journal of Gynaecology and Obstetrics. (2013) 120, no. 1, 3–9, 10.1016/j.ijgo.2012.07.003, 2-s2.0-84871720098, 22959631. 22959631

[56] Tiruye T. Y. , Chojenta C. , Harris M. L. , Holliday E. , and Loxton D. , Intimate Partner Violence Against Women and Its Association With Pregnancy Loss in Ethiopia: Evidence From a National Survey, BMC Women′s Health. (2020) 20, no. 1, 10.1186/s12905-020-01028-z, 32887604. PMC7650300 32887604

[57] Sadore A. A. , Gebretsadik L. A. , and Hussen M. A. , Compliance With Iron-Folate Supplement and Associated Factors Among Antenatal Care Attendant Mothers in Misha District, South Ethiopia: Community Based Cross-Sectional Study, Journal of Environmental and Public Health. (2015) 2015, no. 1, 781973, 10.1155/2015/781973, 2-s2.0-84956880546. 26839573 PMC4709613

