# Supplementary material for: The Association Between Intimate Partner Violence and Anemia Among Ethiopian Women in Their Reproductive Age (15–49 Years): Analysis of National Survey Data
Source: Biomed Res Int. 2025 Sep 22;2025:6321439. doi: 10.1155/bmri/6321439 (PMC12451215; doi:10.1155/bmri/6321439)
Supplement: Supplementary file 1 — Supporting Information File S1 Additional supporting information can be found online in the Supporting Information section. summarizes the definitions and categories of potential confounding variables adjusted in our multivariable models to assess the independent association between intimate partner violence and anemia. [file BMRI-2025-6321439-s001.docx]

**Supplementary file 1** List of control variables, definitions and their categories

| Variable | Measurement and/or categories |
| --- | --- |
| Age of respondent (in years) | The age of the woman categorized as 15-19, 20-24, 25-29, 30-34, 35-39, 40-44, 45-49 |
| Age at first marriage | <15 years, 15-18years, $\geq$ 18 years |
| Educational status | Maximum educational level categorized as uneducated, primary and secondary+ |
| Employment status | Employed/not employed based on their response to “have you been employed in the last 12 months” |
| Place of residence | Rural, urban |
| Region | Region of residence: 11 administrative regions of Ethiopia during the time of the survey |
| Decision-making autonomy | Coded as ‘yes’ if she reported being involved in all decisions regarding her own health care, major household purchases and visits to her family or relatives. |
| Contraceptive use | Women’s contraceptive use status categorized as: never used, used |
| BMI (kg/m^2^) | <18.5, 18.5 -24.9, $\geq$ 25 |
| Number children ever born | Number of children women ever had grouped as one or less, 2-3, 4-5 and 6 or more |
| Substance abuse | Classified ‘yes’ if respondent reported drinking any alcohol, khat chewing or smoking and ‘no’ otherwise. ‘Khat’ is a green leafy vegetable consumed as a stimulant. |
| Socioeconomic status (household wealth index) | Measured based on the number and kind of goods households have and housing characteristics (drinking water, toilet facility, flooring material and availability of electricity), and was generated using principal component analysis and classified into quintiles from 1 (very poor) to 5 (very rich). |
| Access to media | If respondent reportedly read a newspaper, listened to the radio, or watched television; categorized as No, less than once a week, at least once a week. |
